# The Prognostic Significance of Pretreatment Serum CEA Levels in Gastric Cancer: A Meta-Analysis Including 14651 Patients

**DOI:** 10.1371/journal.pone.0124151

**Published:** 2015-04-16

**Authors:** Kai Deng, Li Yang, Bing Hu, Hao Wu, Hong Zhu, Chengwei Tang

**Affiliations:** 1 Department of Gastroenterology, West China Hospital, Sichuan University, Chengdu, Sichuan, China; 2 Department of Abdominal Cancer, West China Hospital, Sichuan University, Chengdu, Sichuan, China; IPO, Portuguese Institute of Oncology of Porto, PORTUGAL

## Abstract

**Background:**

Carcinoembryonic antigen (CEA) is commonly used as a serum tumor marker in clinical practice; however, its prognostic value for gastric cancer patients remains uncertain. This meta-analysis was performed to assess the prognostic value of CEA and investigate CEA as a tumor marker.

**Methods:**

PubMed, EMBASE and other databases were searched for potentially eligible studies. Forty-one studies reporting the prognostic effect of pretreatment serum CEA expression in gastric cancer patients were selected. Data on 14651 eligible patients were retrieved for the meta-analysis. Based on the data extracted from the available literature, the hazard ratio (HR) and 95% confidence interval (CI) for an adverse prognosis were estimated for gastric cancer patients with elevated pretreatment serum levels of CEA (CEA+) relative to patients with normal pretreatment CEA levels (CEA-).

**Results:**

The CEA+ patients had a significantly poorer prognosis than the CEA- patients in terms of overall survival (OS: HR 1.716, 95% CI 1.594 - 1.848, *P*< 0.001), disease-specific survival (DSS: HR 1.940, 95% CI 1.563 - 2.408, *P*< 0.001), and disease-free survival (DFS: HR 2.275, 95% CI 1.836 - 2.818, *P*< 0.001). Publication bias and an influence of different cut-off values were not observed (all *P*> 0.05). In the pooled analyses of multivariate-adjusted HRs, the results suggested that pretreatment serum CEA may be an independent prognostic factor in gastric cancer (OS: HR 1.681, 95% CI 1.425 - 1.982; DSS: HR 1.900, 95% CI 1.441 - 2.505; DFS: HR 2.579, 95% CI 1.935 - 3.436).

**Conclusion/Significance:**

The meta-analysis based on the available literature supported the association of elevated pretreatment serum CEA levels with a poor prognosis for gastric cancer and a nearly doubled risk of mortality in gastric cancer patients. CEA may be an independent prognostic factor for gastric cancer patients and may aid in determining appropriate treatment which may preferentially benefit the CEA+ patients.

## Introduction

Gastric cancer is one of the most common gastrointestinal cancers worldwide, and millions of patients die of this disease each year. Currently, the survival rate for gastric cancer is still unsatisfactory (20–25%), especially in developing countries[1]. This fact may be partly attributable to the late diagnosis of gastric cancer. In addition to TNM stage and choice of treatment, the prognosis of gastric cancer patients may be affected by other factors such as tumor differentiation and behavior and genetic abnormalities[2,3,4]. Therefore, the prognostic prediction for gastric cancer patients is very important in the selection of a suitable treatment strategy.

Gold and Freedman identified carcinoembryonic antigen (CEA) in 1965[5]. CEA has sialofucosylated glycoforms that serve as selectin ligands and facilitate the metastasis of colon carcinoma cells[6,7,8]. It is produced in a high proportion of carcinomas in many other organs[9]. CEA plays a role in tumor metastasis, which greatly affects the prognosis, and it may be partly associated with gastric cancer prognosis. A systemic review of serum markers for gastric cancer reported that elevated CEA levels were found in patients with gastric cancer and were associated with patient survival[10]. Many studies have supported preoperative CEA levels as predictors for the prognosis of gastric cancer[11,12,13,14,15,16,17,18,19]. However, other studies have reported the opposite results[20,21,22,23,24,25]. Thus, the conflicting results have led to confusion regarding the prognostic value of pretreatment CEA levels in patients with gastric cancer. Controversy remains regarding the prognosis of gastric cancer patients with increased CEA levels.

Thus, we performed a meta-analysis based upon the published literature to analyze the association between pretreatment CEA levels and risk of mortality in gastric cancer, to consider data from the conflicting studies together, and to estimate the prognostic value of elevated pretreatment serum levels of CEA in gastric cancer patients.

## Materials and Methods

### Search strategy

We performed a systemic search for all relevant literature. PubMed was searched with the following index formula: [("Stomach Neoplasms"[Mesh]) AND "Carcinoembryonic Antigen"[Mesh]) AND (("Survival Rate"[Mesh]) OR ("Prognosis"[Mesh])]. EMBASE was searched by using the following formula: gastric AND cancer AND CEA AND ('prognosis'/syn OR 'prognosis') AND [humans]/lim. The Journal of Clinical Oncology (JCO), American Society of Clinical Oncology (ASCO) annual meeting and the Cochrane Library were manually searched. All potentially relevant publications were retrieved and evaluated in detail. Cited references in the eligible studies were scanned for any other relevant studies. These searches for published articles were augmented with the searches for unpublished reports. The latest search update was on November 20, 2014.

### Study selection

**Fig 1:**
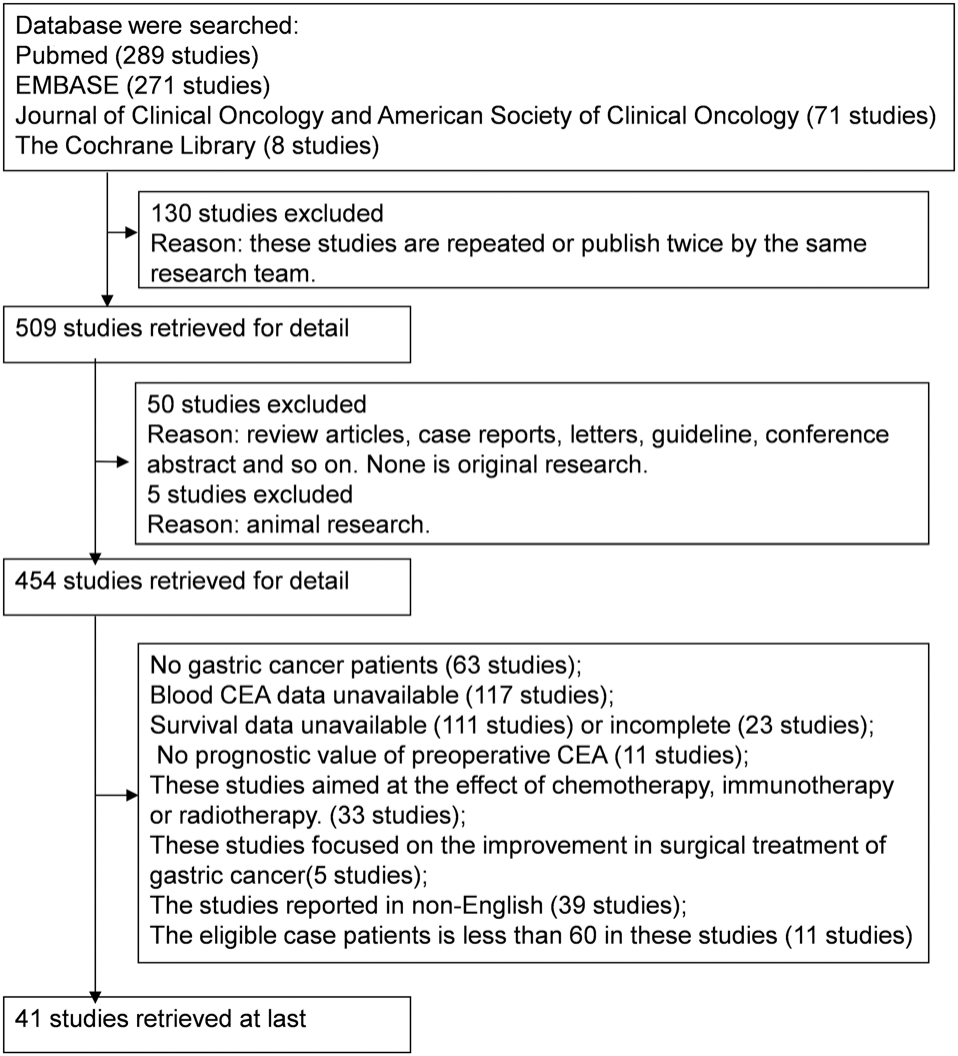
Flow chart of the meta-analysis.

All articles retrieved in the systemic search were independently assessed by two reviewers (Kai Deng and Chengwei Tang) for eligibility using the following inclusion criteria: (i) all participating patients were histologically diagnosed with gastric carcinoma; (ii) studies included pretreatment CEA levels in blood; and (iii) the hazard ratio (HR) for adverse prognosis for patients with elevated pretreatment levels of CEA(CEA+) versus those with normal CEA levels (CEA-) could be extracted from multivariate Cox’s hazards proportional analysis, Kaplan-Meier survival curves or log-rank tests available in the papers. The exclusion criteria were as follow: (i) non-original research articles (such as reviews, comments, letters, conference abstracts, case reports); (ii) a small data set (eligible patients < 60); (iii) studies aimed at the effect of chemotherapy, immunotherapy, radiotherapy or novel treatment; (iv) studies published in non-English languages; and (v) the required data were not available. The flow chart for study selection is shown in Fig 1. If the data sets overlapped or were duplicated, those articles with more information were retained. For articles written by the same authors or that reported results obtained from the same series of patients in multiple publications were identified, the largest or the most informative study was retained.

### Data abstraction

In accordance with the inclusion and exclusion criteria, the studies retrieved from the initial search were screened independently by two researchers (Kai Deng and Chengwei Tang). All selected studies were observational in design because it was impossible to randomly assign patients to CEA+ or CEA- groups. A standardized data extraction protocol was applied to each paper, from which the following data were extracted: first author, publication year, study period, cut-off value, number of CEA+/CEA- cases, number of eligible cases, gender, age, tumor stage, follow-up period, extent of resection, hazard ratio (HR), 95% confidence interval (CI), and covariates adjusted in multivariate Cox’s hazards proportional regression analyses. The HR and 95% CI values were extracted directly or indirectly from each eligible study. If the HR and its 95% CI value were not presented directly, they were estimated from the corresponding data provided in the articles using the statistical methods reported previously[26]. Regarding overlapped or duplicated data set, four studies that were reported by Duraker N et al.[20,27] and Yamashita K et al.[28,29] respectively, were found. The two studies[29,20] with a longer follow-up period or larger sample size were retained. The 9-star Newcastle-Ottawa Scale was applied In the quality assessment of the included studies (non-randomized studies)[30].

### Statistical analyses

Overall survival (OS), disease-specific survival (DSS) and disease-free survival (DFS) were chosen as gastric cancer outcomes for this meta-analysis. These values were calculated from the time of diagnosis until the time of death from all causes, death from gastric cancer, recurrence or last follow-up visit. In the studies involving the independent prognostic value of serum CEA levels, HRs and its 95%% CIs values were calculated from the multivariate Cox proportional hazards regression analysis. For studies that referred to univariate survival analysis, the HRs and 95% CIs values were estimated from survival curves or the variance and its *P*-value (the log-rank test) adopting a series of steps[26]. The presence of CEA- was used as the reference category in the meta-analysis (CEA+ vs. CEA-).

For the mixture of log-rank and multivariate Cox model estimates published in studies, prognostic effects were combined adopting a fixed-effects or random-effects model. Statistical heterogeneity among the included studies was assessed with the *I* ^*2*^ statistic (significance at 5% level)[31]. If the heterogeneity was insignificant, a fixed-effects model with an inverse variance method was chosen [32]. If the heterogeneity was observed, the following procedure was applied to explain it: (i) subgroup analysis or (ii) sensitivity analysis to investigate the sources of heterogeneity. After excluding the studies that potentially biased the results, pooled analyses were performed; (iii) a random-effects model with the DerSimonian-Laird method[33] was applied if the above methods had failed. A meta-regression analysis was performed to assess the extent of heterogeneity derived from the study characteristics (i.e., gender composition, serosal involvement, rate of curative surgery, lymph node involvement rate, proportion of stage III-IV and CEA-positive rate). The mean differences in HRs for CEA in gastric cancer among studies with variant characteristics were evaluated in the meta-regression analyses. Meta-regression was performed using the “metareg” command in Stata statistical software. In addition, the potential publication bias was assessed with a Begg’s funnel plot and Egger’s test in the meta-analysis (significance at 5% level)[34]. Statistical analysis was performed using Stata 12.0 software (StataCorp LP, College Station, TX). All *P*-values were two-sided, and significance was assumed at the 5% level. The HRs and 95% CIs are shown as forest plots (the sizes of the squares are proportional to the weight of each study).

## Results

### Study inclusion and characteristics

We found 639 relevant studies with the systemic search. After careful screening and assessment, 41 studies that met the criteria were identified. The eligible cases of the included studies totaled 14651 patients [the eligible cases of some studies [35,24,36,21,37] that appeared more than once in the meta-analysis for various endpoints of outcomes (i.e., OS, DSS or DFS), were counted only one time]. The characteristics of the included studies are summarized in Table 1. In accordance with the Newcastle-Ottawa scale, the quality score of the included studies ranged from 6 to 9 (S1 File).

**Table 1:** Baseline characteristics of the included studies.

Author, Publish year	Female/Male	Serosal involvement^a^ (+/-)	Curative resection/palliative treatment	Lymph node involvement (-/+)	TNM stage I+II/III+IV	Cut-off value	CEA(+)/CEA(-) (No. of eligible patients)	Follow-up period (months)	Outcome, HR (95% CI), Data extraction, The covariates adjusted for
Cetin B, 2005[36]	31/39	50/20	34/36	7/63	11/59	10 ng/ml	21/49 (70)	24(7–45)	DFS 1.45 (0.76–2.75), OS 1.90 (0.97–3.71), estimated from variance and the *P*-value, non-adjusted.
Aloe S, 2003[61]	72/94	116/50	NA	61/105	60/106	5 ng/ml	39/127 (166)	36.7(2.7–125.7)	DFS 1.93 (1.24–3.02), Estimated from variance and the *P*-value, non-adjusted.
Gaspar MJ, 2001[21]	27/55	68/14	77/5	23/59	27/55	5 ng/ml	13/69 (82)	36	DFS 4.33 (1.81–10.37), Estimated from variance and the *P*-value, non-adjusted; OS 0.80 (0.30–2.50), Cox regression model, Adjusted for Age, Localization, Tumor stage, Histological, CA19-9 and CA72-4.
Kim DH, 2011[62]	174/305	NA	479/0	352/127	411/68	7 ng/ml	11/468 (479)	60.7(9.8–84.8)	DFS 1.37 (0.47–3.94), Estimated from variance and the *P*-value, non-adjusted.
Nakagoe T, 2002[59]	74/144	62/156	185/33	113/105	136/82	2.5 ng/ml	40/178 (218)	62.3(1.3–117.3)	DSS 1.44 (0.77–2.70), Cox regression model; Adjusted for Age, Gender, size, location, Borrmann type, histology, TNM stage and CA19-9, SLX.
Louhimo J, 2004[60]	73/73	NA	78/68	NA	40/106	5 ng/ml	27/119 (146)	>24	DSS 1.47 (0.89–2.43), Estimated from variance and the *P*-value, non-adjusted.
Marrelli D, 1999[16]	58/95	80/73	115/38	60/93	78/75	5 ng/ml	32/121 (153)	>60	DSS and OS 2.23 (1.51–3.30), Extracted from survival curves, non-adjusted.
Ucar E, 2008[24]	32/63	79/16	NA	23/72	28/67	5 ng/ml	23/72 (95)	36	DSS and OS 1.43 (0.37–2.50), Cox regression model, Adjusted for Age, Localization, TNM stage, histology, CA19-9, CA72-4 and AFP.
Tachibana M, 1998[15]	60/136	64/132	NA	118/78	132/64	5 ng/ml	29/167 (196)	60–120	DSS 4.51 (2.00–10.15), Cox regression model, Adjusted for Nodal involvement, depth of invasion, Lauren classification, size, operation type, Borrmann type, CA19-9 and AFP.
Yamashita K, 2008^b^[29]	119/263	NA	382/0	11/371	275/107	2.5 ng/ml	51/331 (382)	60	DSS 1.72 (1.09–2.70)0, Cox regression model, Adjusted for TNM stage, age, ND40, CA199 and vascular invasion.
Yamashita K, 2008^c^[29]	42/65(3 missed)	68/39 (3 missed)	110/0	5/102	0/107 (3 missed)	2.5 ng/ml	22/88 (110)	60	DSS 2.02 (1.14–3.56), Extracted from survival curves, non-adjusted.
Chan AO, 2003[35]	NA	NA	NA	NA	31/78 (7 cases excluded)	5 ng/ml	24/56 (80), 36 cases missed	36	DSS and OS 2.66 (1.65–4.30), Extracted from survival curves, non-adjusted.
Victorzon M, 1995[54]	NA	NA	56/44	NA	39/61	3 ng/mg	30/70 (100)	60	OS 1.50 (1.04–2.17), Extracted from survival curves, non-adjusted.
Ishigami S, 2001[42]	165/384	0/549	463/86	281/268	NA	10 ng/ml	103/446 (549)	42 (12–76)	OS 1.70 (1.00–2.80), Cox regression model, Adjusted for Nodal involvement, depth of invasion, size, lymphatic invasion and CA19-9.
Nakane Y, 1994[13]	323/542	584/281	627/238	383/482	498/367	5 ng/ml	249/616 (865)	NA	OS 1.52 (1.18–1.97), Cox regression model, Adjusted for location, Borrmann type, size, depth of invasion, Nodal involvement, peritoneal metastasis, liver metastasis, curability and histology.
Nakajima K, 1998[23]	Male/Female = 2.1/1	16/82 (12 missed)	NA	73/23 (14 missed)	75/33 (2 missed)	4.6 ng/ml	24/82 (106), 4 missed	36	OS 0.82 (0.29–2.32), Extracted from survival curves, non-adjusted.
Reiter W, 1997[52]	NA	NA	55/48	NA	46/57	4 ng/ml	28/75 (103)	60	OS 1.34 (0.51–3.56), Extracted from survival curves, non-adjusted.
Ikeguchi M, 2009[25]	25/65	30/60	NA	50/40	53/37	5 ng/ml	17/73 (90)	37 (3–76)	OS 1.06 (0.50–2.24), Cox regression model, Adjusted for TNM stage, CA19-9, CRP, IL-6 and IL-10.
Kim DY, 2000[17]	109/216	195/130	NA	152/173	170/155	5 ng/ml	94/231 (325)	60	OS 1.84 (1.25–2.71), Extracted from survival curves, non-adjusted.
Duraker N, 2001[20]	52/116	119/49	NA	54/114	68/100	5 ng/ml	NA (145)	50	OS 1.27 (0.93–1.75), Cox regression model, Adjusted for Gender, age, location, size, histology, depth of invasion, lymph node metastasis, liver metastasis and CA19-9.
Kodera Y, 1996[41]	231/432	NA	566/94 (3 missed)	391/242 (30 missed)	471/192	50 ng/ml	110/553 (663)	NA	OS 1.48 (0.93–2.35), Cox regression model; Adjusted: gender, location, Borrmann type, histopathology, CA19-9 and TNM stage.
Dilege E, 2010[55]	28/47	2/73 (T_1_ 4,T_2_ 28, T_3_ 41,T_4_ 2)	75/0	14/60 (1 cases missed)	30/45	5 ng/ml	25/50 (75)	60	OS 1.26 (0.74–2.16), Extracted from survival curves, non-adjusted.
Xia H.H.-X., 2009[38]	36/61	20/77 (T_1_ 6,T_2_ 23, T_3_ 48,T_4_ 20)	46/51	14/83	28/69	5 ng/ml	59/38 (97)	60	OS 2.65 (1.48–4.72), Cox regression model, Adjusted for age, gender, MIF, size, differentiation, TNM stage and operability.
Nakata B, 1998[40]	29/67	29/67	NA	78/18 (N0-1/N2)	71/25	6.5 ng/ml	NA (96)	50	OS 1.38 (0.27–7.09), Cox regression model, Adjusted for peritoneal metastasis, hepatic metastasis, depth of invasion, lymph node metastasis, lymphatic invasion, venous invasion, sIL-2R, CA19-9 and PBMC number.
Kochi M, 2000[39]	130/355	98/317	Curability A,176; B,138; C,67 (JCGC)	235/162	256/213 (16 missed)	5 ng/ml	92/393 (485)	100	OS 1.94 (1.02–3.70), Cox regression model, Adjusted for CA19-9, Age, location, gross type, TNM stage, depth of invasion, histological type, Nodal involvement, lymphatic invasion, venous invasion and curability.
Zhang YH, 2009[51]	50/116	125/41	NA	50/116	62/104	5 ng/ml	12/64 (76), 90 missed	60	OS 3.02 (1.61–5.65), Extracted from survival curves, non-adjusted.
Takahashi I 1994[14]	NA	131/218	278/70 (1 unknown)	171/178	197/152	5 ng/ml	32/317 (349)	60	DSS 4.34 (2.86–6.59), Extracted from survival curves, non-adjusted.
Park HS 1998[43]	86/117	NA	59/28(Aim of surgery: curative 59, palliative 12, bypass 6)	NA	0/203	5 ng/ml	99/72 (171)	>30	OS 1.78(1.20–2.64), Cox regression model, Adjusted for age, performance status, metastasis pattern, bone involvement, peritoneal seeding, lung metastasis and liver metastasis.
Staab HJ 1982[12]	121/269	NA	139/206 (radical resection 139, palliative 141, unresectable 65)	NA	72/259 (44 cases were excluded)	4 ng/ml	108/237 (345)	60	OS 1.85 (1.42–2.40), Extracted from survival curves, non-adjusted.
Wang CS 1994[53]	457/865	911/411	961/361	911/411	529/793	5 U/dl	254/1029 (1283), 39 cases missed	60	OS 1.61 (1.36–1.89), Extracted from survival curves, non-adjusted.
Koga T 1987[11]	182/286	249/124	419/49	164/209	212/207	20 ng/ml	73/346 (468), 419 cases were shown in the survival curve	60	OS 3.77 (2.94–4.84), Extracted from survival curves, non-adjusted.
Migita K 2013[46]	146/402	NA	Resection: R0 535, R1 13	344/204	423/125	5 ng/ml	145/396 (541), 7 case missed	Median 45.1, 5-years	OS 1.75 (1.18–2.59), Cox regression model, Adjusted for Age, sex, diabetes mellitus, chronic renal failure, preoperative chemotherapy, tumor depth, lymph node metastasis, distant metastasis, respectability, CA199, postoperative complication and prognostic nutritional index.
Liu X 2012[44]	81/192	NA	D2 gastrectomy + first and second tier lymph nodes	77/196	49/224	10 ng/ml	44/229 (273)	median 61.2	OS 2.809 (1.823–4.327), Cox regression model, Adjusted for CA199, CA50, Tumor size, pN stage and Nervous invasion.
Jiang X 2012[45]	553/1157	NA	R0 resection or palliative gastrectomy	NA	1197/513	5 ng/ml	233/1433 (1666)	43.0 (1–123)	OS 1.234 (0.955–1.595), Cox regression model, Adjusted for age, body mass index, tumour location, white cell count, neutrophils, lymphocytes, CA199, tumour stage and mGPS.
Kanetaka K 2013[47]	190/407	121/459	R0 resection 523, R1+2 resection 74	361/236	418/179	5 ng/ml	73/517 (590)	37.4 (0.5–132.8)	OS 0.821 (0.408–1.653), Cox regression model, Adjusted for Tumor size, histologic type, lymphatic invasion, venous invasion, depth of tumor invasion, lymph node metastasis. Adjuvant chemotherapy and CEA in peritoneal lavage.
Kim JG 2013[37]	223/398	16/605	Surgical gastrectomy	311/310	441/180	5ng/ml	57/564 (621)	120	DFS 2.242 (1.561–3.220), Cox regression model, Adjusted for age, stage, NUAK2, PDK-1, pAMPK and MAPK3/1; OS 2.242 (1.561–3.220), Cox regression model, Adjusted for age, stage, NUAK2, PDK-1, pAMPK and MAPK3/1.
Bogenschutz O 1986[56]	Femle/male = 0.526	NA	curative 92, palliative 136	133/135(1 case missed)	NA	5 ng/ml	66/203 (269)	60	OS 2.14 (1.61–2.83), Extracted from survival curves, non-adjusted.
Li F 2013[58]	428/1073	1171/330	potentially curative gastrectomy plus lymphadenectomy and chemotherapy	560/941	623/878	5 ng/ml	329/1172 (1501)	60	OS 1.100 (0.973–1.245), Extracted from survival curves, non-adjusted.
Ogoshi K 1988[57]	69/176	118/127(T_1_ 95,T_2_ 32, T_3_ 53,T_4_ 65)	gastrectomy	NA	NA	7 ng/ml	34/170 (204), 41 cases missed	60	OS 4.176 (2.389–7.301), Extracted from survival curves, non-adjusted.
Zhou F 2013[48]	34/101	NA	curative surgery	NA	39/96	NA	76/59 (135)	39 (3–55)	OS 1.98 (0.75–5.23), Cox regression model, Adjusted for CA199, sex, age, tumor differentiation, TNM stage, Her2 status, primary/metastasis and CRM1 expression.
Ye X.-T. 2014[49]	45/72	75/42 (T_3_ +T_4_ 75, T_1_ +T_2_ 42)	77 total gastrectomy, 40 partial gastrectomy	47/70	47/70	5 ng/ml	35/82 (117)	38 (4–62)	OS 3.279 (2.007–5.357), Cox regression model, Adjusted for TNM stage and NUAK1; DFS 3.269 (2.041–5.237), Cox regression model, Adjusted for NUAK1.
Kim YJ 2014[50]	88/160	NA	Palliative chemotherapy or radiotherapy	NA	NA	5 ng/ml	95/120 (215), 33 cases missed	60	OS 1.420 (1.070–1.880), Cox regression model, Adjusted for ECOG performance status, patient group, peritoneal metastasis, and hypercalcemia.

^a^: If positive/negative serosal data were provided in original articles, the data were extracted directly. If the depth of invasion was not listed in a paper, T_4_ + T_3_ / T_1_ + T_2_ (published before 2009) or T_4_ /T_1_ + T_2_ + T_3_ (published after 2009; T3 and T4 stages were redefined in 2009 UICC TNM stage classification for gastric cancer) were alternatively used.

^b^: Retrospective research.

^c^: Prospective research. OS: overall survival; DSS: disease-specific survival; DFS: disease-free survival; NA: not available.

### Risk of OS

**Fig 2:**
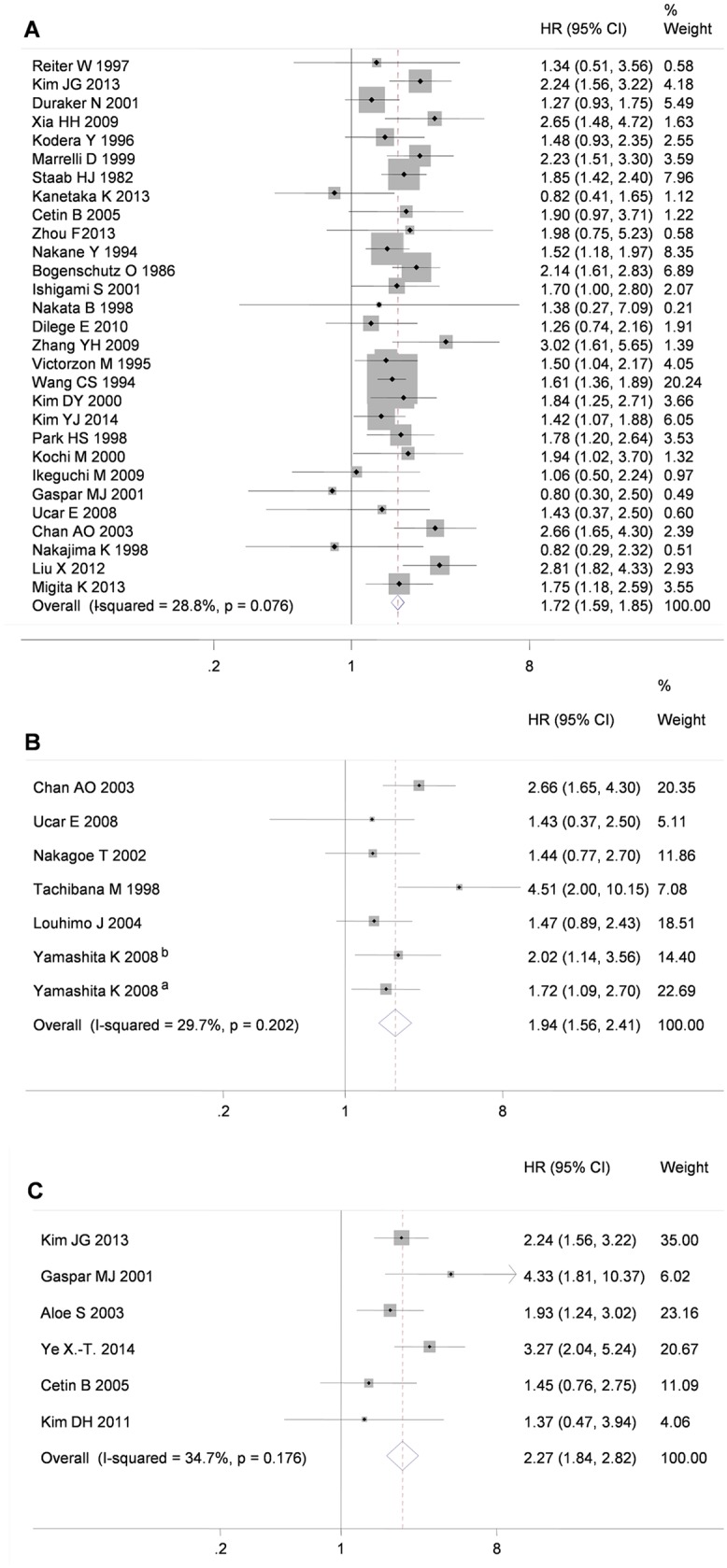
Forest plots for OS (A), DSS (B) and DFS (C) in the CEA+ patients with gastric cancer relative to the CEA- patients are shown. ^a^, Prospective research; ^b^, Retrospective research; The *I* ^*2*^ was used to assess the proportion of total variation in the estimated HRs that was due to between-study heterogeneity. A fixed-effects model was applied for the pooled analysis.

**Fig 3:**
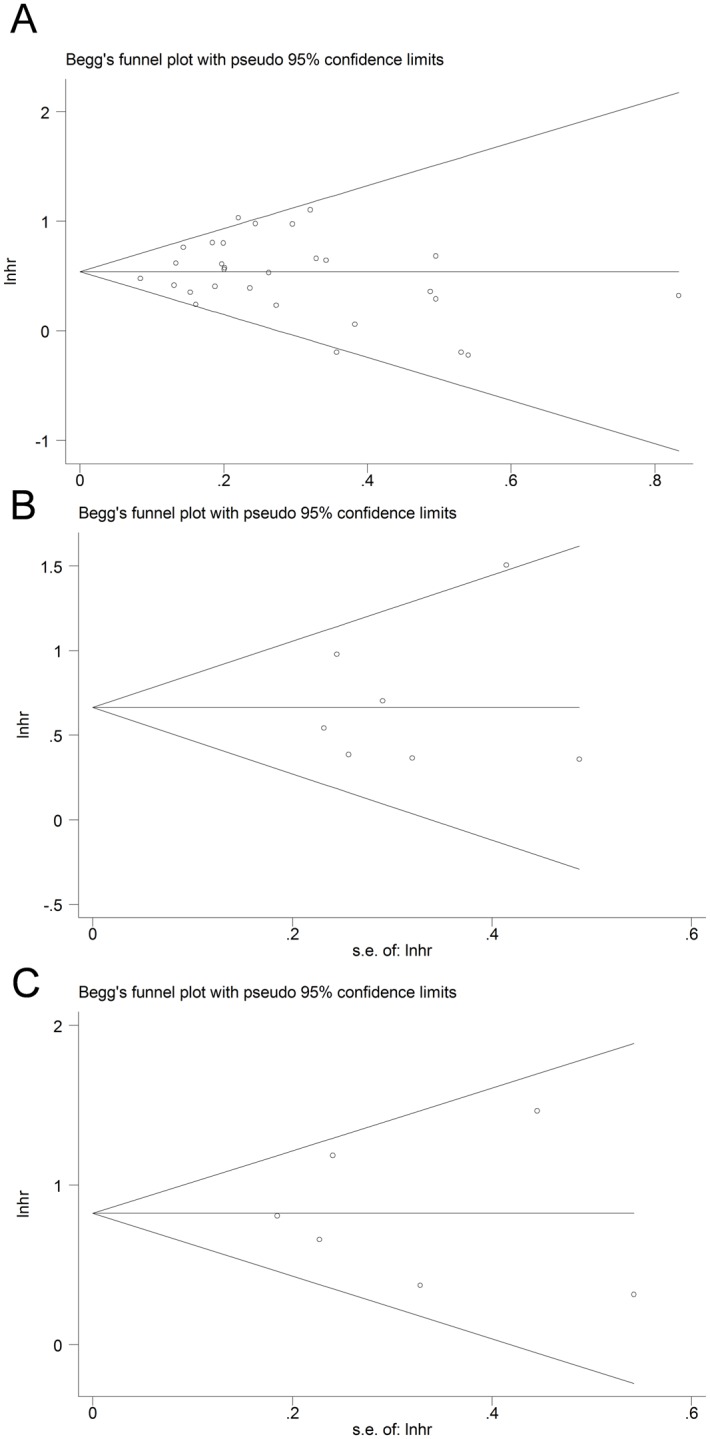
Funnel plots to evaluate publication bias of OS (A), DSS (B) and DFS (C).

The meta-analysis for OS comprised 34 studies including 12605 patients with gastric cancer. HRs and 95% CIs were available in 19 studies[38,39,40,24,41,13,25,42,20,43,21,44,45,46,47,37,48,49,50]. In the remaining studies, the values were extracted from the published survival curves in 14 studies[51,35,52,12,53,54,55,56,17,11,16,57,23,58], and estimated from the variance and its *P*-value (the Log-rank test) from one study[36] using the statistical methods previously reported[26]. The pooled HR and 95% CI values of these 34 studies were estimated (HR 1.786, 95% CI 1.550–2.060), but significant heterogeneity was observed among these studies with respect to OS (*I* ^*2*^ = 77.7%, n = 34, *P*< 0.001: Table 2). The following sensitivity analysis showed that heterogeneity could be attributed mainly to five studies[11,57,45,58,49]. After excluding the five reports, the significant heterogeneity disappeared (Table 2). In the meta-analysis of the remaining 29 studies, the results suggested that the CEA+ patients with gastric cancer had a worse OS than the CEA- patients (HR 1.716, 95% Cl 1.594–1.848; *I* ^*2*^ = 28.8%, *P* = 0.076, n = 29: Fig 2A). No evidence of publication bias was found in the pooled analysis (Begg test *P* = 0.329; Egger’s test *P* = 0.773: Fig 3A). In the following subgroup analysis by cut-off values (CEA >= 5ng/ml versus CEA < 5 ng/ml group), no influence of different the cut-off levels used in the studies was observed (heterogeneity between groups: *P* = 0.720, in Table 2). In the meta-analysis of the excluded studies[11,57,45,58,49], the pooled HR estimate was 2.276 (95%CI, 1.264–4.098,n = 5; I^2^ = 96.1%, *P*< 0.001). A further subgroup analysis was performed to eliminate the heterogeneity among the excluded studies (Table 2). The results indicate that the sample sizes of the included studies might have affected the pooled HR. Although the pooled HR of two studies[45,58] (eligible cases > 1000) was decreased, the conclusion remained unchanged (pooled HR 1.127, 95%CI 1.011–1.258, n = 2, I^2^ = 0.0%).

**Table 2:** Sensitivity analysis and subgroups analyses.

Subgroups	No. of Study	Eligible Sample^a^	Heterogeneity *P*_Q_ ^b^ (*I* ^2^)	HR (95% CI); *P* value	Heterogeneity between subgroups
**Overall Survival**					
all included	34	12511	*P*_*Q*_< 0.001 (77.7%)	1.786 (1.550, 2.059); *P*< 0.001	
with omission^c^	29	8604	*P*_*Q*_ = 0.073 (29.2%)	1.714 (1.592, 1.845); *P*< 0.001	
**The excluded studies**	5	3907	*P*_*Q*_< 0.001 (96.1%)	2.276 (1.264–4.098); *P* = 0.006	
eligible cases < 1000	3	740	*P*_*Q*_ = 0.807 (0.0%)	3.730 (3.034–4.585); *P*< 0.001	*P*< 0.001
eligible cases > 1000	2	3167	*P*_*Q*_ = 0.397 (0.0%)	1.127 (1.011–1.258); P = 0.031	
**Disease Specific Survival**					
all included	8^e^	1576	*P*_*Q*_ = 0.006 (64.7%)	2.226 (1.592, 3.112); *P*< 0.001	
with omission^d^	7^e^	1227	*P*_*Q*_ = 0.202 (29.7%)	1.940 (1.563, 2.408); *P*< 0.001	
**The excluded study**	^1^	349	-	4.340 (2.859–6.588); *P*< 0.001	
**Disease Free Survival**					
all included	6	1535	*P*_*Q*_ = 0.176 (34.7%)	2.275 (1.836, 2.818); *P*< 0.001	
**Subgroup Analysis by Cut-off Value** ^**g**^					
**OS (included studies)**					
>= 5ng/ml	24	7815	*P*_*Q*_ = 0.037 (36.9%)	1.721 (1.590–1.863); *P*< 0.001	*P* = 0.733
< 5 ng/ml	4	654	*P*_*Q*_ = 0.407 (0.0%)	1.656 (1.350–2.032); *P*< 0.001	
**DSS(included studies)**					
>= 5ng/ml	4	437	*P*_*Q*_ = 0.077 (56.2%)	2.169 (1.603–2.935); *P*< 0.001	*P* = 0.302
< 5 ng/ml	3	710	*P*_*Q*_ = 0.736 (0.0%)	1.727 (1.268–2.352); *P*< 0.001	
**DFS(included studies)**					
>= 5ng/ml	6	1535	*P*_*Q*_ = 0.176 (34.7%)	2.275 (1.836, 2.818); *P*< 0.001	-
< 5 ng/ml	-	-	-	-	

^a^, Ineligible cases reported in original articles were excluded

^b^, Q statistic *p*-value

^c^, Omission of five studies[11,57,45,58,49] to which significant heterogeneity could be attributed mainly in accordance with sensitivity analysis

^d^, Omission of one study[14] to which significant heterogeneity could be attributed mainly in accordance with sensitivity analysis

^e^, One study [29] that contained retrospective research and prospective research, was counted twice

^g^: If the cutoff value was unavailable in a study, it was omitted in the meta-analysis. OS, overall survival; DSS, disease-specific survival; DFS, disease-free survival; NA, not available; HR, hazard ratio; CI, confidence interval.

In the following meta-regression analyses, no study characteristics [proportion of serosal invasion, female sex, node-positive status, advanced stage (III-IV TNM stage) or CEA+ cases reported in different papers] were identified as potential confounding factors on the estimated effect (all *P*> 0.05), with the exception of curative treatment (*P* = 0.048, Table 3). This result indicated that the proportion of patients who underwent curative treatment may influence the estimated effect of CEA on mortality in gastric cancer. There is no evidence to support that those study characteristics (except curative treatment) significantly modify the association between preoperative serum CEA level and mortality in patients with gastric cancer.

**Table 3:** Using meta-regression analysis to explore the impact of study characteristics.

		Publish year	Rate of TNM stage III+IV	Rate of lymph node involvement	Rate of curative treatment	Rate of serosal invasion	Rate of female	Rate of CEA(+) case	cutoff value	HR extraction methods
***p*-Value of Meta-regression (eligible studies)**	OS^a^	0.580 (29)	0.162 (27)	0.244 (22)	0.048* (15)	0.768 (19)	0.891 (24)	0.830 (26)	0.707 (27)	0.396 (29)
	DSS^a^	0.222 (7)	0.930 (7)	0.531 (5)	0.559 (4)	0.756 (3)	0.469 (6)	0.603 (7)	0.435 (7)	0.594 (7)
	DFS	0.742 (6)	0.948 (6)	0.951 (6)	0.722 (3)	0.933 (5)	0.214 (6)	0.832 (6)	0.191 (6)	0.373 (6)

^a^: Due to significant heterogeneity among studies, five studies were excluded in the meta-analysis of the OS group, and one study was excluded in the DSS group.

*: *P*< 0.05

### Risk of DSS

The meta-analysis for DSS comprised seven studies including 1576 patients with gastric cancer. HRs and 95% CIs were obtained directly from four studies[29,24,15,59]. In the remaining studies, two sets of values were estimated from the published survival curves[35,14], and one sets of values was calculated from the variance and its *P*-value[60]. The HRs and 95% CIs of these eight trials (one study contained a retrospective research and a prospective research)[29] were pooled (HR 2.226, 95% CI 1.592–3.112) and significant heterogeneity was observed among these studies regarding DSS (*I* ^*2*^ = 64.7%, n = 8; *P* = 0.006, in Table 2). In the subsequent sensitivity analysis, we identified one study[14] that contributed the most to heterogeneity. After removing the study, the heterogeneity disappeared (*I* ^*2*^ = 29.7%, n = 7, *P* = 0.202; Table 2). In a meta-analysis of the remaining seven trials, the results suggested that the CEA+ patients with gastric cancer had a higher mortality risk than the CEA- patients (HR 1.940, 95% CI 1.563–2.408; Fig 2B). No evidence of publication bias was found (Begg test, *P* = 0.881; Egger’s test: *P* = 0.716; Fig 3B). In the subsequent subgroup analysis of cut-off values, no influence of various cut-off values used in the studies was detected (heterogeneity between groups: *P* = 0.302; Table 2).

In the subsequent meta-regression analysis, no study characteristics [proportion of female, serosal invasion, curative resection, lymph node involvement, CEA+ cases or advanced stage (TNM stage III-IV) reported in different papers] were found to be major sources of heterogeneity (all *P*> 0.05, Table 3). This result indicated that these characteristics were not associated with the prognostic effect of pretreatment CEA levels for DSS in gastric cancer patients.

### Risk of DFS

The meta-analysis for DFS comprised six studies including 1535 patients with gastric cancer. HRs and 95% CIs were directly obtained from two studies [37,49], and the other values were estimated from the variance and the *P*-value[61,36,21,62]. In the meta-analysis for DFS, the CEA+ patients with gastric cancer suffered higher risks of recurrence than the CEA- patients (HR 2.275, 95% CI 1.836–2.818), and no significant heterogeneity was found among studies (*I* ^*2*^ = 34.7%, n = 6, *P* = 0.176; Fig 2C and Table 2). No evidence of publication bias was found (Begg test *P* = 0.573; Egger’s test *P* = 0.897; Fig 3C).

In the subsequent meta-regression analysis, no study characteristics [proportion of female, serosal invasion, curative resection, lymph node involvement, advanced stage (TNM stage III-IV) or CEA+ cases reported in different papers] were identified as the major sources of heterogeneity (all *P*> 0.05, Table 3). No association between clinical status and the prognostic effect of preoperative CEA levels for DFS was found in gastric cancer.

### Covariate adjustment and subgroup analysis

In a multivariate Cox’s proportional regression analysis of the included studies, the multivariate-adjusted HRs were adjusted by stratification factors (e.g., stage of disease, performance status and other prognostic factors and so on) at randomization[63]. The multivariate-adjusted HRs and 95% CIs were directly obtained from 20 studies [38,39,40,24,41,13,25,42,20,43,21,44,45,46,47,37,48,49,29,50]. Based on the multivariate-adjusted HRs, the meta-analyses were performed in terms of OS, DSS and DFS.

The meta-analysis of these multivariate-adjusted HRs showed that the CEA+ gastric cancer patients suffered poorer prognosis than the CEA- patients (for OS [38,39,40,24,41,13,25,42,20,43,21,44,45,46,47,37,48,49,50], HR 1.631, 95% CI 1.462–1.820, n = 17; for DSS [29,24,15,59], HR 1.900, 95% CI 1.441–2.505, n = 5; for DFS [37,49], HR 2.579, 95% CI 1.935–3.436, n = 2). The HRs adjusted for similar variables were pooled (listed in Table 4). The stratified analyses of the multivariable adjusted HRs were performed only if there were at least 3 eligible studies. After covariate adjustment, the studies that were adjusted for having the same clinical status were combined to estimate the prognostic effect of pretreatment serum CEA (Table 4). In subgroup analyses, the results suggested that patient characteristics (i.e., age, Borrmann type, CA199, depth of invasion, sex, histology, liver metastasis, location, nodal involvement, TNM stage, tumor size, lymphatic invasion, and peritoneal metastasis) were not associated with the prognostic effect of CEA on OS, DSS or DFS in patients with gastric cancer. These results provided evidence to support pretreatment serum CEA levels as possibly being an independent prognostic factor for adverse outcomes in patients with gastric cancer.

**Table 4:** Subgroup analyses of multivariate-adjusted HRs.

Outcome	Adjusted variable	No. of Studies with adjusted HR^a^	Pooled HR	95% CI	I^2^	*p*-Value Heterogeneity^b^	*p*-Value meta analysis
**OS**							
	all included	17	1.631	1.462–1.820	30.40%	*0*.*114*	*<0*.*001*
**Adjusted for**	age	9	1.727	1.471–2.027	21.00%	*0*.*256*	*<0*.*001*
	Borrmann type	3	1.522	1.256–1.918	0.00%	*0*.*769*	*<0*.*001*
	CA199	11	1.613	1.371–1.898	73.80%	*0*.*263*	*<0*.*001*
	Depth of invasion	7	1.476	1.261–1.728	0.00%	*0*.*502*	*<0*.*001*
	sex	4	1.819	1.407–2.351	0.00%	*0*.*485*	*<0*.*001*
	histology	7	1.391	1.178–1.642	0.00%	*0*.*531*	*<0*.*001*
	Liver metastasis	4	1.482	1.242–1.768	0.00%	*0*.*617*	*<0*.*001*
	Location	6	1.435	1.210–1.702	0.00%	*0*.*732*	*<0*.*001*
	Nodal involvement	8	1.592	1.373–1.846	45.50%	*0*.*076*	*<0*.*001*
	TNM stage	8	1.823	1.476–2.250	15.30%	*0*.*309*	*<0*.*001*
	Tumor size	6	1.668	1.232–2.259	67.00%	*0*.*01*	*<0*.*001*
	Lymphatic invasion	4	1.47	1.046–2.046	18.70%	*0*.*297*	*0*.*027*
	Venous invasion	3	1.313	0.833–2.070	36.40%	*0*.*208*	*0*.*241*
	Peritoneal metastasis	4	1.531	1.286–1.821	0.00%	*0*.*845*	*<0*.*001*
**DSS**							
	all included	5	1.9	1.441–2.505	29.50%	*0*.*225*	*<0*.*001*
**Adjusted for**	Age	3	1.593	1.130–2.244	0.00%	*0*.*879*	*0*.*008*
	CA199	4	1.864	1.359–2.557	46.60%	*0*.*132*	*<0*.*001*
	TNM stage	3	1.593	1.130–2.244	0.00%	*0*.*879*	*0*.*008*
**DFS**							
	all included	2	2.579	1.935–3.436	35.40%	*0*.*214*	*<0*.*001*

^a^: If the number of included studies were equal to or greater than 3, the pooled analysis of HRs adjusted for the same covariate were conduced.

^b^: *p*-value for the Cochrane Q test of heterogeneity within a subgroup.

If a *p*-value was less than 0.05, a random-effects model was be used. Otherwise, a fixed-effects model was chosen.

## Discussion

The general consensus is that pretreatment serum CEA levels are associated with an adverse prognosis in colon cancer[64,65,66]. It is known that high serum CEA levels are closely associated with tumor load. Currently, CEA is one of the most commonly used biomarkers in clinical practice. Whether pretreatment serum CEA levels have a prognostic value for the survival of patients with gastric cancer is still disputed[23,24].

Previous studies have provided contradictory evidence on the prognostic value of pretreatment serum CEA levels in gastric cancer[15,23,55,60]. The inconsistent views can be partly explained by the limited number of eligible cases and the limited statistical power of a single study. The results reported in most studies have shown a tendency for the CEA+ patients with gastric cancer to have a higher risk of mortality than the CEA- patients. Hideaki Shimada et al. recognized the issue and published a review regarding serum markers to partly support the prognostic value of CEA in gastric cancer[10]. However, due to limitations on the length and content of the article, the risk of an adverse prognosis was not quantized, and some different views were not pooled for the estimated value of CEA in gastric cancer. Therefore, in the present study, a formal meta-analysis was performed to provide a quantitative summary of the existing evidence and a general evaluation of the prognostic prediction ability in gastric cancer patients according to pretreatment serum CEA levels.

With a meta-analysis, the number of eligible patients on the basis of similar endpoints can be enlarged, and the lower statistical power in studies can be overcome. Based on the available data, a meta-analysis can strengthen statistical power, narrow the 95% CI and integrate different views on prognostic effects of pretreatment serum CEA levels in gastric cancer. A meta-analysis can provide more knowledge regarding CEA in gastric cancer.

The publication year of the included studies ranged from 1982 to 2014. The lengthy time period led to great differences in the study characteristics from one institution to another (Table 1), which might have contributed to most of the heterogeneity in the pooled analyses. Despite different follow-up periods, cutoff values, ethnicities and treatments used in the included studies, these confounding factors might be randomly balanced across the CEA+ and CEA- groups. In addition, the study characteristics (i.e., tumor characteristics and physical condition) that varied greatly across studies might have influenced the effect size estimate for risk of mortality in patients with gastric cancer. Meta-regression analyses were conducted to confirm that most study characteristics (i.e., serosal invasion, female sex, lymph node involvement or advanced stage reported in different papers) had no significant effect on the pooled HR estimates.

Moreover, the exclusion of studies mainly aimed at the effect of chemotherapy, radiotherapy, immunotherapy or novel therapy reduced the confounding factors with varied treatments, and only observational studies with similar endpoints were selected for the meta-analysis. In addition to OS as an endpoint for survival assessment, DSS and DFS were introduced to eliminate interference from other causes of mortality in the meta-analysis. In the meta-analysis, the prognostic effects of pretreatment serum CEA on OS (HR 1.716, 95% CI 1.594–1.848), DSS (HR 1.940, 95% CI 1.563–2.408) and DFS (HR 2.275, 95% CI 1.836–2.818) in patients with gastric cancer were confirmed (Fig 2). It is intriguing that the average effects for DSS and DFS were higher than that for OS. This result indicates that pretreatment serum CEA levels in gastric cancer patients can provide predictive information regarding other outcomes.

Finally, gastric cancer patients prognosis can be mainly affected by performance status and tumor characteristics. The multivariate-adjusted HRs reported in the studies were controlled for potential confounding factors. Then, the pooled the multivariate-adjusted HRs to confirm that serum CEA levels were associated with prognosis independently from other prognostic factors (Table 4). In the subsequent subgroup analyses, the HRs that were adjusted for the same patient characteristics were pooled to minimize the effect of each covariate. The independent prognostic value of pretreatment serum CEA levels remains in patients with gastric cancer after adjustment for covariates (i.e., age, Borrmann type, CA199, depth of invasion, sex, histology, liver metastasis, location, nodal involvement, TNM stage, tumor size, lymphatic invasion, peritoneal metastasis; shown in Table 4).

To our knowledge, this meta-analysis aiming to summarize the prognostic effect of pretreatment CEA levels in patients with gastric cancer is one of relatively few that have been reported. In this study, a significant difference in prognosis was confirmed between pretreatment CEA+ and CEA- patients with gastric cancer for all stratified analyses. The results showed that increased pretreatment serum CEA levels nearly doubled the risk of mortality in patients with gastric cancer.

### Limitations

The prognostic effect of serum CEA levels on OS and DSS might be interpreted with caution because of the significant heterogeneity among the studies. To reduce the heterogeneity among the studies, we conducted a sensitivity analysis and removed the studies that contributed most to the heterogeneity. The significant between-study heterogeneity was then eliminated in the subsequent meta-analysis (Table 2). The absence of publication bias and heterogeneity provided more evidence for the maintenance of substantial consistency in the results across the eligible studies. We could not exclude the possibility of residual confounding by uncontrolled factors. However, the pooled multivariate-adjusted HRs for OS, DSS and DFS showed that the prognostic effect of pretreatment serum CEA levels persisted even after adjustment for multiple potential confounders. Therefore, pretreatment serum CEA levels are likely independently associated with prognosis in patients with gastric cancer. However, this hypothesis needs to be validated by large-scale, prospective clinical studies.

### Conclusions

This meta-analysis of currently available studies provides sufficient evidence to confirm that the pretreatment serum CEA level is likely an independent prognostic predictor for gastric cancer patients. This result suggests that clinicians should consider CEA levels. The CEA+ patients are likely to suffer a worse prognosis and would therefore benefit more from intensive neoadjuvant therapy compared with CEA- patients. Further clinical trials with the standardized methodology and criteria are required for confirmation.

## Supporting Information

S1 File The Newcastle-Ottawa Scale (NOS) for Assessing the Quality of Studies Included. (XLS)

S2 File The Original Data Extracted from Studies Included. (XLS)

S1 Fig PRISMA 2009 Flow Diagram in this meta-analysis. (DOC)

S1 Table PRISMA 2009 checklist in this meta-analysis. (DOC)

S2 Table The search results of relevant articles. (DOC)
