# Supplementary material for: The Prognostic Significance of Pretreatment Serum CEA Levels in Gastric Cancer: A Meta-Analysis Including 14651 Patients
Source: PLoS One. 2015 Apr 16;10(4):e0124151. doi: 10.1371/journal.pone.0124151 (PMC4400039; doi:10.1371/journal.pone.0124151)
Supplement: S1 Fig — (DOC) [file pone.0124151.s003.doc]

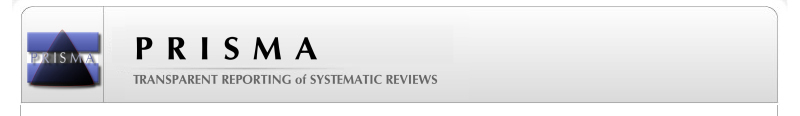
**PRISMA 2009 Flow Diagram**

**Screening**

**Included**

**Eligibility**

**Identification**

Records identified through database searching
(n = 560)

Additional records identified through other sources
(n = 79)

Records after duplicates removed
(n = 509)

Records screened
(n = 509 )

Records excluded
(n = 55)

Full-text articles assessed for eligibility
(n = 454)

Full-text articles excluded, with reasons
(n = 413)

Studies included in qualitative synthesis
(n = 41)

Studies included in quantitative synthesis (meta-analysis)
(n = 41)
