# Supplementary material for: The Prognostic Significance of Pretreatment Serum CEA Levels in Gastric Cancer: A Meta-Analysis Including 14651 Patients
Source: PLoS One. 2015 Apr 16;10(4):e0124151. doi: 10.1371/journal.pone.0124151 (PMC4400039; doi:10.1371/journal.pone.0124151)
Supplement: S2 Table — (DOC) [file pone.0124151.s005.doc]

S2 Table. The search results of relevant articles

| The lastest search (2014.09.13) | No. | Searches | Results |
| --- | --- | --- | --- |
| Pubmed | 1# | "Stomach Neoplasms"[Mesh] | 74750 |
|  | 2# | "Carcinoembryonic Antigen"[Mesh] | 13612 |
|  | 3# | "Survival Rate"[Mesh] | 126298 |
|  | 4# | "Prognosis"[Mesh] | 1108500 |
|  | Total | 1# and 2# and (3# or 4#) | 289 |
| Embase | 1# | gastric AND cancer | 84906 |
|  | 2# | CEA | 41472 |
|  | 3# | 'prognosis'/syn OR 'prognosis' | 620920 |
|  | 4# | [humans]/lim | 15242532 |
|  | Total | 1# and 2# and 3# and 4# | 271 |
| Cochrane library | Total | CEA and gastric cancer and survival     search in title, abstract or keyword | 8 |
| JCO and ASCO | Total | Searching gastric cancer and CEA and prognosis (all words) in full text | 71 |
